# Prostaglandin E2 increases migration and proliferation of human glioblastoma cells by activating transient receptor potential melastatin 7 channels

**DOI:** 10.1111/jcmm.13931

**Published:** 2018-10-19

**Authors:** Yafei Tian, Tingting Yang, Shuntai Yu, Cuiyun Liu, Min He, Changlong Hu

**Affiliations:** ^1^ Department of Physiology and Biophysics School of Life Sciences Institutes of Brain Science Fudan University Shanghai China

**Keywords:** glioblastoma cell, migration and proliferation, PGE2, PKA, TRPM7

## Abstract

Recent studies showed that both prostaglandin E2 (PGE2) and transient receptor potential melastatin 7 (TRPM7) play important roles in migration and proliferation of human glioblastoma cells. In this study, we tested the association between PGE2 and TRPM7. We found that PGE2 increased TRPM7 currents in HEK293 and human glioblastoma A172 cells. The PGE2 EP3 receptor antagonist L‐798106 abrogated the PGE2 stimulatory effect, while EP3 agonist 17‐phenyl trinor prostaglandin E2 (17‐pt‐PGE2) mimicked the effect of PEG2 on TRPM7. The TRPM7 phosphotransferase activity‐deficient mutation, K1646R had no effect on PGE2 induced increase of TRPM7 currents. Inhibition of protein kinase A (PKA) activity by Rp‐cAMP increased TRPM7 currents. TRPM7 PKA phosphorylation site mutation S1269A abolished the PGE2 effect on TRPM7 currents. PGE2 increased both mRNA and membrane protein expression of TRPM7 in A172 cells. Knockdown of TRPM7 by shRNA abrogated the PGE2 stimulated migration and proliferation of A172 cells. Blockage of TRPM7 with 2‐aminoethoxydiphenyl borate (2‐APB) or NS8593 had a similar effect as TRPM7‐shRNA. In conclusion, our results demonstrate that PGE2 activates TRPM7 via EP3/PKA signalling pathway, and that PGE2 enhances migration and proliferation of human glioblastoma cells by up‐regulation of the TRPM7 channel.

## 1 INTRODUCTION

Prostaglandin E2 (PGE2), derived from arachidonic acid through the action of cyclooxygenase‐1/‐2 (COX‐1/‐2) and PGE2 synthase, is a potent endogenous lipid mediator.^1^ It is the most widely produced prostanoid in the human body.^2^ PGE2 plays important roles in various pathological and physiological functions, such as cell proliferation,^3^ apoptosis,^4^ cancer,^5^, ^6^ inflammation,^7^ hypertension,^8^ Alzeheimer's disease,^9^ diabetes^10^, ^11^ and immune response.^12^ PGE2 functions through activation of four specific G‐protein‐coupled receptors (GPCR) subtypes, termed EP1, EP2, EP3 and EP4.^2^ The EP1 receptor couples to Gαq and causes an intracellular Ca^2+^ increase. The EP2 and EP4 receptors couple to Gαs to increase intracellular cAMP formation, while the EP3 receptor couples to Gαi to decrease intracellular cAMP production.^2^

The transient receptor potential melastatin 7 (TRPM7) is a cation channel with a functional serine/threonine protein kinase domain in its C‐terminal.^13^, ^14^ TRPM7 is ubiquitously expressed and plays important roles in various pathological and physiological processes, including Mg^2+^ homeostasis, cell proliferation, embryonic development and differentiation, cancer, ischemic stroke, cardiovascular and neurodegenerative diseases (for review, see reference 15, 16). Inhibition of TRPM7 has been extensively studied. TRPM7 can be inhibited by intracellular proton, Ba^2+^, Sr^2+^, Mn^2+^, Zn^2+^, Mg^2+^ and Mg‐nucleotide,^14^, ^17^, ^18^ by PIP2 depletion^19^ and by sphingosine.^20^ However, how TRPM7 is stimulated remains largely unknown. TRPM7 inward currents can be increased by acidic pH,^21^ and TRPM7 has also been shown to be modulated by mechanical stimuli.^22^, ^23^

Glioblastoma is the most common and aggressive type of brain tumors. Recent studies suggested that both PGE2 and TRPM7 played key roles in proliferation, migration and invasion of human glioblastoma cells.^4^, ^24^, ^25^, ^26^, ^27^, ^28^ Previous studies suggested that TRPM7 could be modulated by G‐protein‐coupled receptors.^19^, ^29^ As PGE2 exerts its biological effects by G‐protein‐coupled receptors, we hypothesise that there is a crosstalk between PGE2 and TRPM7 in the regulation of proliferation and migration of glioblastoma cells.

In this study, we tested the effect of PGE2 on TRPM7 overexpressed in HEK293 cells and endogenous TRPM7 in A172 glioblastoma cells, and whether PGE2 increases proliferation and migration of human glioblastoma cells by stimulation of TRPM7.

## 2 MATERIALS AND METHODS

### 2.1 Cell culture and transfection

HEK293 (human embryonic kidney cells) and human glioblastoma A172 cells were purchased from the cell bank of Chinese Academy of Sciences (Shanghai, China). Cells were cultured in Dulbecco's modified Eagle's medium (DMEM; GIBICO, Grand Island, NY, USA) supplemented with 10% fetal bovine serum and 1% antibiotic antimycotic solution at 37°C with 5% CO_2_. Transient transfections with the murine TRPM7 plasmid and mutant channels were performed using jetPRIME reagents (Polyplus‐transfection) according to the manufacturer's instructions. The cells were used for patching experiments 48 hours after transfection.

### 2.2 Chemicals

17‐pt‐PGE2 was purchased from Cayman chemical. PGE2, 2‐Acetylhydrazide‐10(11H)‐carboxylic acid, 8‐Chloro‐dibenz [b, f]^1^, ^4^ oxazepine‐10(11H)‐carboxylic acid (SC19220), (4Z)‐7‐[(rel‐1S,2S,5R)‐5‐((1,1′‐Biphenyl‐4‐yl)methoxy)‐ ‐2‐(4‐morpholinyl)‐3‐oxocyclopentyl]‐4‐heptenoic acid hemicalcium salt (AH23848), (2E)‐N‐[(5‐bromo‐2‐methoxyphenyl)sulfonyl]‐3‐[2‐(2‐naphthalenylmethyl)phenyl]‐2‐propenamide (L‐798106), 6‐isopropoxy‐9‐xanthone‐2‐carboxylic acid (AH6809), Dibutyryl‐cAMP (Db‐cAMP), Rp‐cAMP (Rp‐Adenosine 3′,5′‐cyclic monophosphorothioate triethylammonium salt) and 2‐APB were purchased from Sigma‐Aldrich. N‐[(1R)‐1,2,3,4‐Tetrahydro‐1‐naphthalenyl]‐1H‐benzimidazol‐2‐amine hydrochloride (NS8593) was purchased from Tocris (Bristol, UK).

### 2.3 RNA extraction and quantitative real‐time polymerase chain reaction

Total RNA was extracted (RNeasy mini kit; QIAGEN, Shanghai, China) and reverse transcribed (2 μg RNA). Real‐time PCR was performed in 20 μL reactions containing: 2 μL of template, 0.4 μmol/L of each paired primer, and SYBR Green Polymerase Chain Reaction master mix. The thermocycling conditions were 94°C, 10 minutes; 38 cycles of 94°C, 30 seconds; 55°C, 30 seconds; 72°C, 30 seconds; and 72°C, 8 minutes. Results were normalised by β‐actin mRNA. Data were calculated by 2^−ΔΔCt^ method and reported as fold change over control. The primers used for real‐time polymerase chain reaction are: forward, 5′‐CTACCGAAGACACTCAT‐3′, reverse, 5′‐ACTCTATTTTGGCACAG‐3′.

### 2.4 Electrophysiology

Whole‐cell currents were recorded using an Axonpatch 200B amplifier (Axon Instruments, Union City, CA, USA). The bath solution contained (in mmol/L): 145 NaCl, 5 KCl, 10 HEPES, 2 CaCl_2_, 10 glucose, pH 7.4 (adjusted with NaOH). The internal solution contained (in mmol/L): 145 Cs‐methanesulfonate, 10 EGTA, 8 NaCl, and 10 HEPES (pH adjusted to 7.3 using CsOH). Recording pipettes (capillary tubing, BRAND) had resistances of 3‐6 MΩ under solution conditions. All recordings were performed at room temperature. Currents were sampled at 10 kHz and filtered at 2 kHz. Drugs were applied by gravity perfusion.

### 2.5 Plasmids and lentiviral production

The shRNA hairpin sequences were inserted into MluI‐ClaI sites of pLVTHM targeting vector, and lentivirus was produced in the 293T cells using a packaging vector psPAX2 and an envelope plasmid pMD2.G. as previously reported.^30^ Oligonucleotides specifying the shRNA are: 5′‐GCAGCAGAGCCCGATATTA‐3′ and scramble, 5′‐AGATCGGCGATCGAACTCA‐3′. Lentivirus infection was performed 24 hours after seeding A172 cells to complete medium. Cells were used for various tests 72 hours after infection.

Murine TRPM7 plasmid was kindly provided by Dr. Lixia Yue.^20^ Site‐directed K1646R, S1264A and S1269A mutageneses were achieved in the TRPM7 channel by using the QuikChange XL Site‐directed Mutagenesis kit (Stratagene, La Jolla, CA, USA). All mutations were confirmed by sequencing.

### 2.6 Protein kinase A (PKA) phosphorylation assay

HEK293 cells were transfected with plasmids for TRPM7 channels 24 hours before PKA phosphorylation assay. Cells were treated with/without 5 μM PGE2 for 10 minutes. Cells were suspended in cold PKA extraction buffer (25 mM Tris‐HCl, pH 7.4, 0.5 mM EDTA, 0.5 mM EGTA, 10 mM β‐mercaptoethanol, and 1% protease inhibitor cocktail) and homogenised using a cold homogeniser. The lysate was centrifuged at 4°C at 14 000 *g* for 5 minutes and the supernatant was analysed by a PepTag assay kit (Promega, Madison, WI, USA) for PKA activity according to the manufacturer's instruction. Phosphorylated and non‐phosphorylated peptide bands were separated on a 0.8% agarose gel and quantitated by scanning densitometry using the ChemiDoc XRS+ System and Image Lab software (Bio‐Rad, Hercules, CA, USA). PKA activity was expressed as the ratio of phosphorylated peptides to the total amount of PepTag A1 peptides loaded.

### 2.7 Cell migration and proliferation assay

A172 cell mobility was tested by wound healing and transwell assay. The wound was made by the culture insert (ibidi). Cells were cultured in DMEM without serum. Average percentage of area closure was measured 24 hours after wounding by ImageJ. Transwell inserts with 8 μm pores in 24 well plates (Corning, Corelle City, NY, USA) were used for the cell immigration assays. 5 × 10^4^ A172 cells were seeded into the upper chambers with/without various treatments in serum‐free DMEM, while the bottom chambers were filled with 800 μL DMEM with 10% FBS. After incubated for 24 hours, cells were washed with PBS for three times, fixed with 4% paraformaldehyde for 30 minutes, and stained with crystal violet staining solution (Beyotime, Shanghai, China) for 4 hours. Cells on the upper surface were removed and the migrating cells on the lower surface of the inserts were photographed and counted by ImageJ. Cell proliferation was tested by the Cell Counting Kit‐8 according to the manufacturer's instructions. Briefly, cells were cultured in 96 well plates in a concentration of 2 × 10^4^ cells/mL, and optical density (OD) of cells was measured at 450 nm after 24 hours.

### 2.8 Biotinylation assay

A172 cells were treated by PGE2 for indicated time, and cell surface proteins were biotinylated subsequently with 0.25 mg/mL Sulfo‐NHS‐SS‐Biotin (Thermo Scientific, Waltham, MA, USA) for 45 minutes at 4°C. Biotinylation was stopped using 50 mM Tris (pH 8.0) for 20 minutes at 4°C. The cells were then lysed with HEPES‐NP40 lysis buffer. Biotinylated proteins were pulled down using Streptavidin Agarose Resin (Thermo Scientific) at 4°C overnight. The surface proteins were washed by lysis buffer for four times and eluted by 1× SDS loading buffer. The membrane proteins were incubated at 50°C for 20 minutes, and used for western blotting.

### 2.9 Western blot

A172 cells were treated with 5 μM PGE2 for 24 hours. Cells were lysed with HEPES‐NP40 buffer (20 mM HEPES, 150 mM NaCl, 0.5% NP‐40, 10% Glycerol, 2 mM EDTA, and 1% Protease Inhibitor Cocktail (Sigma, Munich, Germany), pH 7.5) on ice for 45 minutes. After centrifugation at 12 000 *g* for 15 minutes, the supernatants were mixed with 2× SDS loading buffer and incubated at 95°C for 5 minutes. The protein samples were separated using 8% SDS‐PAGE and transferred to polyvinyldifluoride (PVDF) membranes (Millipore, Burlington, MA, USA). After blocked with 10% nonfat milk in TBST for 1.5 hours at room temperature, the membranes were incubated with primary antibody [Anti‐TrpM7, 1:500 (University of California, Davis); anti‐GAPDH, 1:1000 (Beyotime), Anti‐ATP1A1, 1:800 (Proteintech, Chicago, IL, USA)] in Immunoreaction Enhancer Solution for primary antibody (TOYOBO, Osaka, Japan) overnight at 4°C. The membranes then washed with 0.3‰ TBST for three times and incubated with HRP‐conjugated anti‐mouse IgG (1:1000; Beyotime) for 2 hours at room temperature. Chemiluminescent signals were developed using enhanced chmiluminescence (ECL) reagents (Bio‐Rad) and detected using the ChemiDoc XRS+ System (Bio‐Rad). Image Lab software (Bio‐Rad) was used for quantification of immunoblotting data.

### 2.10 Statistics

Data analysis was performed with Clampfit 10.2 (Axon Instruments) and Origin 8.0 software (OriginLab, Northampton, MA, USA). Statistical analysis consisted of unpaired or paired Student *t* tests. Data are given as means ± SEM, n indicates the number of tested cells or independent tests. *P* < 0.05 was considered statistically significant. Multiple comparisons were analysed using a one‐way ANOVA followed by post hoc Tukey testing.

## 3 RESULTS

### 3.1 PGE2 increases TRPM7 channel currents in HEK293 cells

**Figure 1:**
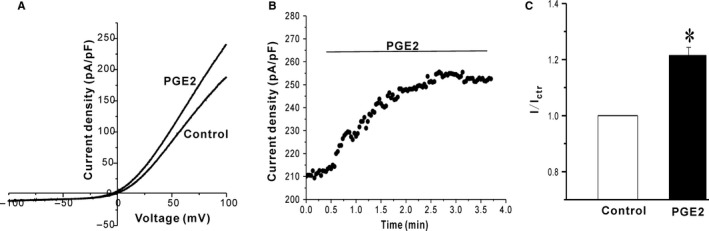
PGE2 increases TRPM7 channel currents in HEK293 cells. (A) Representative current traces show the effect of the extracellular applying of 5 μM PGE2 on TRPM7 channels expressed in HEK293 cells. (B) The time course of the TRPM7 current stimulation by 5 μM PGE2. (C) Statistical analysis of the stimulatory effect of 5 μM PGE2 on TRPM7 at +100 mV. *P < 0.05 compared to vehicle control

We first tested whether PGE2 could regulate the activity of TRPM7 channels. Murine TRPM7 channels were overexpressed in HEK293 cells. The TRPM7 current was elucidated by a ramp protocol from −100 mV to +100 mV. 3‐5 minutes was waited after break in, until the current was stable, and then the drug solution was perfused until a stable simulation level was achieved. As shown in Figure 1, 5 μM PGE2 significantly increased TRPM7 outward currents (fold increase of the TRPM7 currents at +100 mV: 1.22 ± 0.02, n = 13, *P* < 0.05). The stimulatory effect of PGE2 on TRPM7 currents appeared gradually and reached a maximum around three minutes (Figure 1B).

### 3.2 PGE2 increases TRPM7 channel currents via EP3 receptors

**Figure 2:**
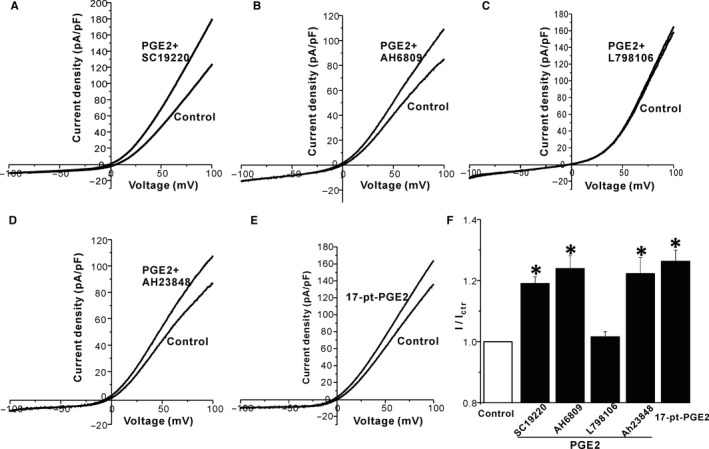
PGE2 increases TRPM7 channel currents by the EP3 pathway. (A‐D) Sample current traces show the effects of SC19220 (EP1 antagonist), AH6809 (EP2 antagonist), L‐798106(EP3 antagonist) and AH23848 (EP4 antagonist) on PGE2 induced stimulation of TRPM7 channels overexpressed in HEK293 cells. (E) Sample current traces show the effect of 17‐pt‐PGE2 (EP3 agonist) on PGE2 induced stimulation of TRPM7 channels. (F) Bar graph plots of means ± SEM of normalised TRPM7 current density. *P < 0.05 compared to control

**Figure 3:**
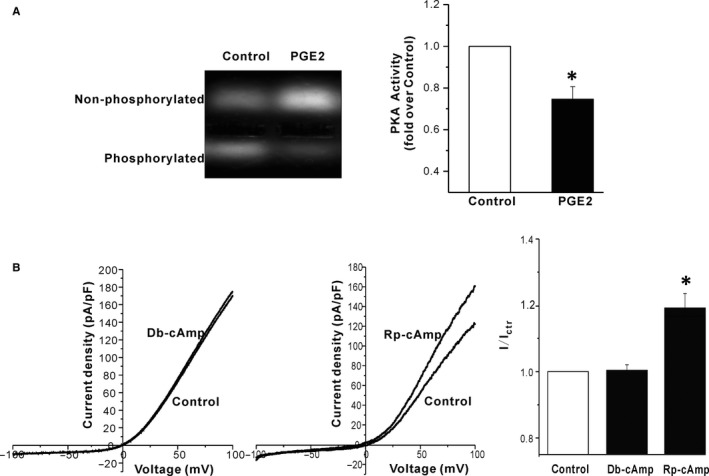
Inhibition of PKA activity increases TRPM7 currents in HEK293 cells. (A) A representative image of PepTag assay measuring activity of cAMP‐dependent PKA in response to PGE2 (left); Bar graph plots of means ± SEM of normalised PKA activity (right). *P < 0.05 compared to control. (B) Representative current traces show the effect of 20 μM Rp‐cAMP on TRPM7 channels (left); Representative current traces show the effect of 20 μM Db‐cAMP on TRPM7 channels expressed in HEK293 cells (middle); Bar graph plots of means ± SEM of normalised TRPM7current density (right). *P < 0.05 compared to control

PGE2 exerts its biological effects through four receptors, termed EP1, EP2, EP3, and EP4. We used SC19220, AH6809, L‐798106, and AH23848 to block the four receptors, respectively. SC19220, AH23848, L‐798106, and AH6809 per se had no effect on TRPM7 channels. 20 μM SC19220, AH6809 and AH23848 did not alter the stimulatory effect of 5 μM PGE2 on TRPM7 channels (fold increase: PGE2+ SC19220: 1.18 ± 0.03, n = 4; PGE2+ AH6809: 1.24 ± 0.05, n = 6; PGE2+ AH23848: 1.21 ± 0.03, n = 8; *P* > 0.05 compared to PGE2 alone, Figure 2). However, 20 μM L‐798106 abrogated the PGE2 induced increase of TRPM7 currents (Figure 2). Moreover, 17‐pt‐PGE2 (20 μM), the EP3 receptor agonist, mimicked the stimulatory effect of PGE2 (fold increase: 1.26 ± 0.04, n = 5). The above data suggested that PGE2 stimulated TRPM7 channels via EP3 receptors. Through EP3 receptors, PGE2 decreases the amount of intracellular cAMP concentration and subsequently inactivates PKA. Therefore, we tested whether PEG2 decreased PKA activity in the HEK293 cells overexpressing TRPM7 channels. As shown in Figure 3A, 5 μM PGE2 reduced the PKA activity in HEK293 cells by 25%. Next, we used Db‐cAMP (a PKA activator) and Rp‐cAMP (a PKA inhibitor) to test whether a PKA‐dependent pathway was involved in the PGE2 effect on TRPM7 channels. As shown in Figure 3, 20 μM Db‐cAMP did not alter the activity of TRPM7 channels, however, 20 μM Rp‐cAMP significantly increased the TRPM7 currents. The data indicated that PGE2 increased TRPM7 currents by reducing PKA activity.

### 3.3 PGE2 increases TRPM7 channel currents by reducing PKA phosphorylation of S1269 site

**Figure 4:**
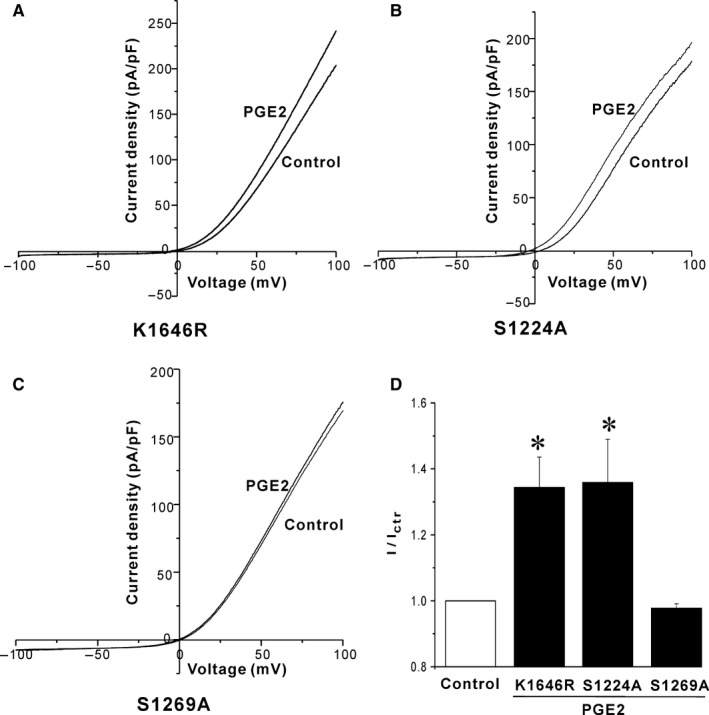
PGE2 increases TRPM7 channel currents by reducing PKA phosphorylation of S1269 site. (A) The phosphotransferase activity‐deficient mutation K1646R did not alter the PGE2 induced stimulatory effect on TRPM7. (B, C) Sample current traces show the effects of mutation of PKA phosphorylation sites S1224A and S1269A on PGE2 induced stimulation of TRPM7 channels expressed in HEK293 cells. (D) Statistical analysis of the effects of the three point mutations on the PGE2 induced increase of TRPM7 currents. n = 5‐11, *P < 0.05 compared to control

Previous studies suggested that the kinase domain of TRPM7 may affect channel regulation.^17^, ^31^ Therefore, we tested whether PGE2 also regulated TRPM7 through the kinase domain. As shown in Figure 4A, K1646R mutant, the phosphotransferase activity‐deficient mutant of TRPM7, presented similar PGE2‐induced increase of TRPM7 currents compared the wild‐type. According to the previous mass spectrometry studies on the phosphorylation sites of murine TRPM7 in HEK293 cells, there are two consensus PKA sites (S1224, S1269) on the C terminus of the channel.^32^ Therefore, we tested whether the two sites were involved in the PGE2 effect on TRPM7. S1224A mutation did not alter PGE2 stimulatory effect on TRPM7, while S1269A mutation abrogated the PGE2 effect on TRPM7 (Figure 4). The data suggested that PGE2 increased TRPM7 currents by reducing PKA phosphorylation of S1269.

### 3.4 Effect of PGE2 on endogenous TRPM7 channels in human glioblastoma A172 cells

**Figure 5:**
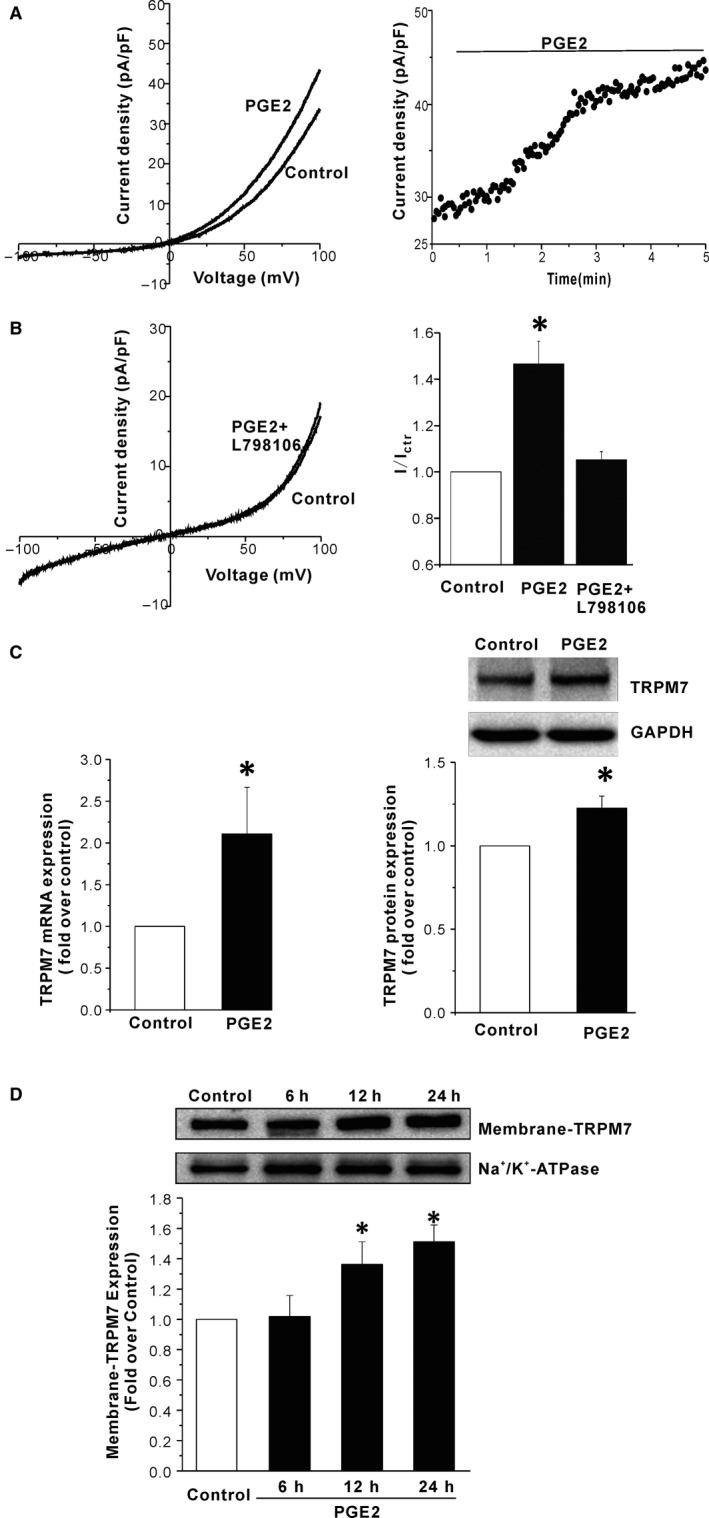
Effect of PGE2 on endogenous TRPM7 channels in human glioblastoma A172 cells. (A) Representative current traces show the effect of 5 μM PGE2 on endogenous TRPM7 channels in A172 cells (left); The time course of the TRPM7 current stimulation by 5 μM PGE2 (right). (B) Sample current traces show the effect of L‐798106 (EP3 antagonist) on PGE2 induced stimulation of TRPM7 channels in A172 cells (left). Bar graph plots of means ± SEM of normalised TRPM7 current density (right). *P < 0.05 compared to control. (C) 24 h incubation with 5 μM PGE2 increased TRPM7 mRNA (left) and protein (right) expression in A172 cells. A representative image (Upper right) of Western blot from four independent experiments. *P < 0.05 compared to control. (D) 5 μM PGE2 increased membrane protein expression of TRPM7 in A172 cells in a time‐dependent manner (n = 5, *P < 0.05)

A172 cells have been shown to express functional TRPM7 channels.^28^ We first investigated whether PGE2 could increase endogenous TRPM7 currents in A172 cells. As shown in Figure 5A, 5 μM PGE2 significantly increased TRPM7 outward currents in A172 cells, and the stimulatory effect was abrogated by EP3 antagonist L‐798106 (fold increase: PGE2: 1.46 ± 0.1, n = 5; PGE2+ L‐798106: 1.05 ± 0.04, n = 4, Figure 5A and B). TRPM7 expression has been shown to be up‐regulated in human glioblastoma,^33^ therefore we next studied the effect of PGE2 on the expression of TRPM7 in A172 cells. As shown in Figure 5C, after 24 hours of treatment with 5 μm PGE2, mRNA expression of TRPM7 was significantly increased by 2.1 fold, and TRPM7 protein expression was increased by 1.23 fold compared to untreated cells. Furthermore, 5 μm PGE2 increased membrane protein expression of TRPM7 in a time‐dependent manner (Figure 5D).

### 3.5 PGE2 enhances migration and proliferation of human glioblastoma cells through stimulating TRPM7 channels

**Figure 6:**
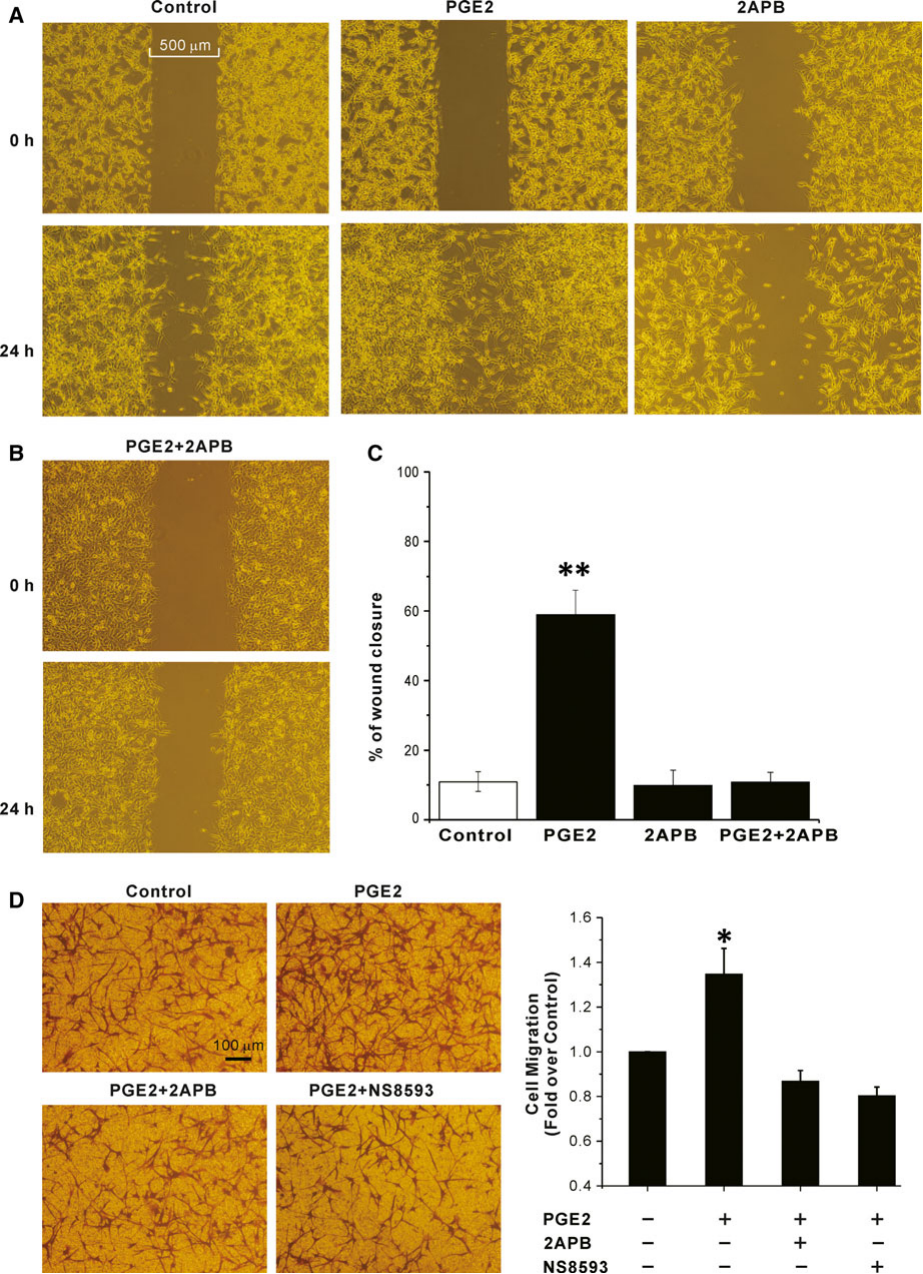
Effects of TRPM7 inhibitor 2‐APB and NS8593 on PEG2 induced migration of A172 cells. (A‐B) Representative images taken at 0 h (top) and 24 h (bottom) after wounding show the effect of 5 μM PGE2 on mobility of A172 cells in the presence/absence of 2‐APB. (C) Statistical analysis of the average percentage of wound closure 24 h after wounding. **P < 0.01 compared to control. (D) Representative images of transwell assays showing migrated A172 cells after 24 h treatment with 5 μM PGE2 in the presence/absence of 2‐APB or NS8593. Statistical analysis of the effects of 2‐APB and NS8593 on the PGE2 induced migration of A172 cells (right, *P < 0.05, n = 3)

**Figure 7:**
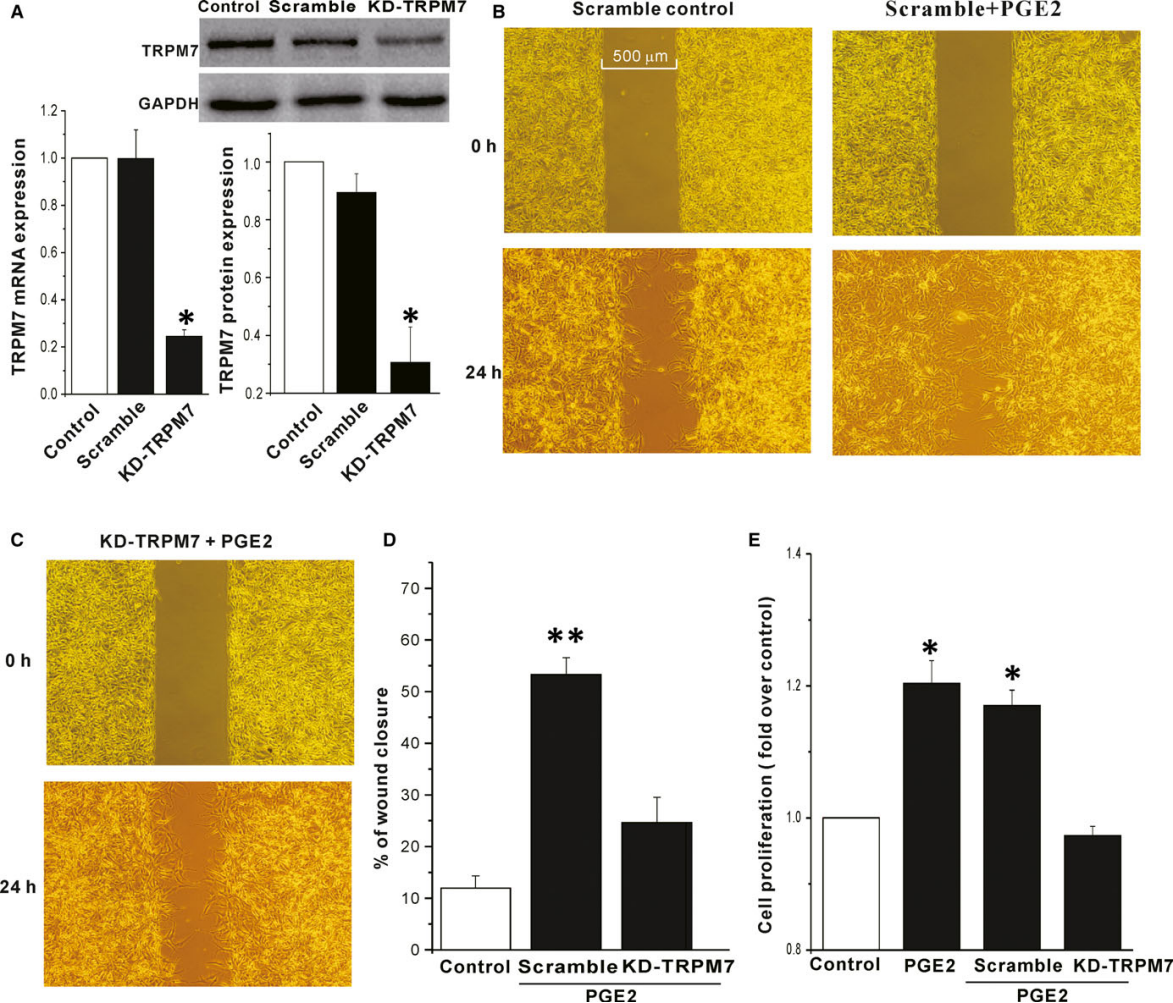
Effect of knockdown of the TRPM7 channel on PEG2 induced migration and proliferation of A172 cells. (A) The mRNA and protein expression of TRPM7 channels was significantly reduced in A172 cells transduced with shRNA‐TRPM7 lentiviruses. A representative image (Upper right) of western blot from three independent experiments. *P < 0.05 compared to scramble control. (B‐C) Representative images taken at 0 h (top) and 24 h (bottom) after wounding show the effect of knockdown of TRPM7 channels on 5 μM PGE2 enhanced mobility of A172 cells. (D) Knockdown of TRPM7 channels abrogated PGE2‐induced migration of A172 cells. **P < 0.01 compared to scramble control (n = 4). (E) 24 h treatment with 5 μM PGE2 significantly increased the proliferation of A172 cell, and knockdown of TRPM7 abrogated the PEG2 induced stimulation (n = 5, *P < 0.05)

The data above suggested that TRPM7 is a downstream target of PGE2 signalling. We then tested whether TRPM7 was involved in the stimulatory effect of PGE2 on migration and proliferation of human glioblastoma cells. We investigated the motility of A172 cells by a wound healing assay. As shown in Figure 6, 24 hours treatment with 5 μm PGE2 significantly increased wound closure of A172 cells, and 100 μm 2‐APB abrogated the stimulatory effect of PGE2 (percent wound closure: control: 11.0 ± 2.8%, PGE2: 58.9 ± 7.1%, PGE2+ 2‐APB: 10.8 ± 2.9%, 2‐APB: 9.8 ± 4.3%, n = 5‐11, *P* < 0.01, Figure 6). The results of wound healing assay were confirmed by using a transwell assay. As shown in Figure 6D, both 2‐APB and NS8593 (10 μm), a recent identified TRPM7 inhibitor,^34^ abrogated the PGE2 induced A172 migration. Moreover, knockdown of TRPM7 using lentiviral vector delivery of shRNA showed similar effects as 2‐APB (Figure 7). Cell proliferation of A172 was tested by a Cell Counting Kit‐8. As shown in Figure 7E, 24 hours incubation with 5 μm PGE2 significantly increased the proliferation of A172 cell, and knockdown of TRPM7 abrogated the PEG2 induced stimulation.

## 4 DISCUSSION

Even though compelling studies show that TRPM7 plays key roles in various pathological and physiological processes, how it is activated under physiological conditions is not fully elucidated. In this study, we found that PGE2 increased TRPM7 currents via EP3/PKA signalling pathway, and that PGE2 enhanced migration and proliferation of human glioblastoma cells by upregulation of TRPM7.

Previous studies showed that TRPM7 could be regulated by Gq‐protein coupled receptors. Activation of muscarinic receptor 1 by carbacol leads to inhibition of TRPM7 overexpressed in HEK293 cells through Gq/PLC.^19^ The mGluR5 agonist (RS)‐2‐chloro‐5‐hydroxyphenylglycine inhibits endogenous TRPM7 currents in hippocampal neurons by Gq/PLC dependent pathway.^14^ TRPM7 in hippocampal neurons is also inhibited by nerve growth factor through PLC‐dependent pathways.^13^ Activation of Gq/PLC signalling pathway by bradykinin, lysophosphatidic acid, or thrombin inhibits TRPM7 overexpressed in N1E‐115 cells when intracellular Mg^2+^ below physiological levels, while stimulates TRPM7 under normal Mg^2+^ levels.^35^

Besides Gq, activation of Gs/i‐coupled receptor has also been shown to regulate TRPM7. Isoproterenol stimulation of Gs‐coupled beta‐adrenergic receptors increases, while carbachol stimulation of Gi‐coupled muscarinic receptors inhibits overexpressed TRPM7 currents in HEK293 cells. The Gs/i‐dependent regulation of TRPM7 is sensitive to intracellular Mg^2+^ levels.^29^ In this study, we showed that PGE2 had a stimulatory effect on TRPM7 by activating Gi‐coupled EP3 receptors. We did not find stimulatory effect of cAMP on TRPM7 since the intracellular Mg^2+^ used is far below physiological level (Figure 3), which is consistent with the previous study.^29^ The kinase domain of TRPM7 has been shown to be required for the channel inhibition by carbachol or Isoproterenol.^29^ However, the PGE2 activation of TRPM7 did not require the kinase activity since the K1646R mutation, which blocked the channel phosphotransferase activity, did not alter the PGE2 induced increase of TRPM7 currents (Figure 4).

S1224 and S1269 sites are located on the coiled‐coil region of C terminus of TRPM7. So far, the function of phosphorylation of the coiled‐coil region remains unclear. Our data suggested that phosphorylation of S1269 suppressed the TRPM7 activity. Based on previous MS‐study S1224 and S1269 sites of TRPM7 are phosphorylated under basal conditions in HEK293 cells.^32^ Our study also suggested that S1269 site is phosphorylated in basal situation since cAMP had no effect on TRPM7 currents, and that could be explained by relative high basal cAMP tone in HEK293 cells.^36^, ^37^

Solid tumors are usually accompanied with inflammation.^38^ PEG2, as a pro‐inflammatory factor, has been shown elevated in several tumors including colon cancer,^39^ prostate cancer,^40^, ^41^ and non‐small cell lung cancer.^42^ Recent studies suggested that PGE2 is also elevated in human glioblastoma and plays a key role in the growth and migration of glioblastoma cells.^24^, ^26^, ^43^ TRPM7 expression has been shown upregulated in human glioblastoma, and contributes to proliferation and migration of glioblastoma cells.^28^, ^33^ Our data demonstrated that TRPM7 is the down streaming target of PGE2 under glioma conditions, as blockage of TRPM7 by 2‐APB or shRNA abrogated the PGE2 induced increase of migration and proliferation of A172 cells. We found that PGE2 increased both mRNA and protein expression of TRPM7 in A172 cells, which could partially explain the upregulation of TRPM7 in human glioblastoma. TRPM7 channel expression has been shown to be up‐regulated by a couple of other endogenous factors including cholesterol, aldosterone and angiotensin II, but none of them has acute effect on channel activity as PGE2 does.^18^, ^44^, ^45^, ^46^

In conclusion, our results provide the first evidence that: (a) PGE2 increases TRPM7 currents by EP3/PKA signalling pathway; (b) PGE2 potentiates TRPM7 channel currents by reducing PKA phosphorylation of S1269 site; (c) PGE2 increases TRPM7 mRNA and protein expression in human glioblastoma cells; (d) PGE2 enhances migration and proliferation of human glioblastoma cells by up‐regulation of TRPM7. Our results reveal a new activation mechanism for TRPM7 channels, and provide further insights into the regulation of migration and proliferation of human gliobastoma cells by PGE2 and TRPM7.

## CONFLICT OF INTEREST

The authors confirm that there are no conflicts of interest.
